# Troubleshooting in Large-Scale LC-ToF-MS Metabolomics Analysis: Solving Complex Issues in Big Cohorts

**DOI:** 10.3390/metabo9110247

**Published:** 2019-10-24

**Authors:** Juan Rodríguez-Coira, María I Delgado-Dolset, David Obeso, Mariana Dolores-Hernández, Guillermo Quintás, Santiago Angulo, Domingo Barber, Teresa Carrillo, María M. Escribese, Alma Villaseñor

**Affiliations:** 1 CEMBIO, Centro de Excelencia en Metabolómica y Bioanálisis, Facultad de Farmacia, Universidad San Pablo CEU, 28668 Madrid, Spain; juan.rodriguezvillanueva@ceu.es (J.R.-C.); maria.delgadodolset@beca.ceu.es (M.I.D.-D.); david.obesomontero@beca.ceu.es (D.O.); may34@comunidad.unam.mx (M.D.-H.); 2 IMMA, Instituto de Medicina Molecular Aplicada, Facultad de Medicina, Universidad San Pablo CEU, 28668 Madrid, Spain; domingo.barberhernandez@ceu.es; 3 Laboratorio de Ensayos de Desarrollo Farmacéutico (LEDEFAR), Facultad de Estudios Superiores Cuautitlán, Universidad Nacional Autónoma de México, Estado de México CP.54714, Mexico; 4 Health and Biomedicine, Leitat Technological Center, 08028 Barcelona, Spain; 5 Analytical Unit, Health Research Institute Hospital La Fe, 46026 Valencia, Spain; 6 Departamento de Matemática Aplicada y Estadística, Universidad San Pablo CEU, 28668 Madrid, Spain; sangulo@ceu.es; 7 Servicio de Alergia, “Hospital Universitario de Gran Canaria, Dr. Negrin”, 35010 Las Palmas de G.C., Spain; tcardia@gobiernodecanarias.org; 8 Departamento de Ciencias Médicas Básicas, Facultad de Medicina, Universidad San Pablo CEU, 28668 Madrid, Spain

**Keywords:** large-scale, metabolomics, LC-QToF-MS, normalization, asthma

## Abstract

Metabolomics, understood as the science that manages the study of compounds from the metabolism, is an essential tool for deciphering metabolic changes in disease. The experiments rely on the use of high-throughput analytical techniques such as liquid chromatography coupled to mass spectrometry (LC-ToF MS). This hyphenation has brought positive aspects such as higher sensitivity, specificity and the extension of the metabolome coverage in a single run. The analysis of a high number of samples in a single batch is currently not always feasible due to technical and practical issues (i.e., a drop of the MS signal) which result in the MS stopping during the experiment obtaining more than a single sample batch. In this situation, careful data treatment is required to enable an accurate joint analysis of multi-batch data sets. This paper summarizes the analytical strategies in large-scale metabolomic experiments; special attention has been given to QC preparation troubleshooting and data treatment. Moreover, labeled internal standards analysis and their aim in data treatment, and data normalization procedures (intra- and inter-batch) are described. These concepts are exemplified using a cohort of 165 patients from a study in asthma.

## 1. Introduction

The application of “omics” sciences for deciphering the mechanisms involved in complex diseases has succeeded, showing a great impact in the recent years not only in the number of publications, but also in the advance of medical treatments [1,2]. Metabolomics has gained attention due to its capability to inquire into the metabolism of living organisms.

Asthma is a prevalent disease that currently affects almost 25% of the population worldwide [3]. The number of cases increases every year, as well as its severity, creating a high economic burden and severely impacting health, especially in children. It is a multifactorial disease in which the clinical phenotypes are not clearly defined; partially because the underlying molecular mechanisms are still largely unknown [3]. In addition, it is associated with secondary comorbidities such as obesity and allergy.

Metabolomics experiments rely on the use of high-throughput analytical techniques, such as nuclear magnetic resonance (NMR) and mass spectrometry (MS) based techniques. The use of high-resolution MS, particularly with a quadrupole-time of flight analyzer coupled to liquid chromatography (LC-QToF-MS), has dramatically increased the sensitivity and specificity of the technique enabling the extension of the metabolome coverage in a single run. Therefore, it obtains a very complete picture of the metabolic fingerprint of a patient.

Usually, the instrumental analysis consists on the measurement of a small number of samples—less than a hundred—in a single batch. However, in large-scale studies samples need to be analyzed across multiple batches. This is required due to different reasons including the shift in the instrument response associated with the contamination of the ionization source after repeated injections of samples. Every time a batch is run, the instrument is cleaned, calibrated and conditioned, leading to minor changes in the analytical response. This introduces a between-batch systematic error that needs to be properly addressed to enable the joint analysis of multi-batch data.

For this purpose, the development of algorithms for a post-acquisition correction of both intra- and inter-batch effects is an active field of research [4,5,6]. On the other hand, additional aspects in large-scale studies, such as the use of internal standards (ISs), the preparation of the quality control samples (QCs), the strategy for sample preparation, the instrumental analysis and the batch design are an integral part of the study design and must be clearly established in advance [7].

The “IS” is a compound (or a group of them) with the closest chemical nature possible to the target metabolites found in the sample and that is not naturally present in it. ISs are widely used in quantitative (targeted) LC-MS methods to assess the effects of the matrix on ion suppression or enhancement, as well as to test the extraction efficiency [8]. In untargeted metabolomic studies, ISs should be carefully selected to avoid interference with the a priori unknown metabolites. Thus, isotopically labeled analogues (often with ^2^H or ^13^C) are frequently selected due to their nearly identical physico-chemical properties to the unlabeled metabolites. The ISs selected should provide a broad coverage of the different classes of metabolites that are expected to be found in the samples. However, their use in untargeted LC-QToF-MS is limited to the assessment of instrument performance, as the IS intensity should not be used to correct between batch systematic errors since the metabolites present in the samples can influence the IS estimates by cross contribution [4,7,9]. Currently, different normalization strategies to correct the instrumental drift have been developed, and most of them rely on the information contented in QCs [10,11,12].

Furthermore, the preparation of the QCs in large-scale studies is not obvious. Ideally, the QC should be prepared by pooling a small volume of all samples; however, in large-scale studies sometimes this is not feasible. Moreover, even when this is possible, if the time of sample thawing for QC preparation is extensive, it may induce the enzymes in blood samples to activate, leading to a change in the original sample. Therefore, alternative strategies have been suggested, i.e., the use of a pool of randomly selected samples that could represent the sample population [13]. Nonetheless, the exact number of samples needed to represent the whole population should be defined beforehand. Additional factors increasing the complexity of large-scale studies have not been addressed, such as if samples should be prepared all at once or in different sets, and how could this impact the retrieved metabolomic profiles. These “naïve” aspects are essential. Additionally, randomization of the complete set of samples into the batches is the statistically correct approach; however, there is no warrant that batches will be accurately normalized after integration. 

Large-scale studies are mostly unavoidable when working with heterogeneous diseases like asthma, where hundreds of samples are collected, analyzed together and later compared to extract useful biological information. In this work, we propose a series of strategies and troubleshooting suggestions for large-scale LC-QToF-MS metabolomic studies. Special attention is given to the experimental design and to methodological checkpoints required to achieve a proper analysis and integration of a large set of samples.

This work exemplifies from begging to the end the process to be followed in a large-scale analysis in metabolomics. We show for the first time the inclusion of a deuterated lysophosphocholine, sphingolipid and fatty acid, apart from carnitine and isoleucine in the IS mix to cover the complete retention time span in a reverse phase analysis, which is one of the most common analysis in metabolomic fingerprinting studies. Regarding this IS mix, we describe its response in a large-scale sequence in different ways, and we show its utility in the analysis. In addition, we present the comparison of different normalization strategies such as IS, total useful signal (TUS), QC-SVRC normalization and QC-norm. The novelty of this work relies on an experimental strategy to prove that a robust normalization is possible in large-scale studies by using replicates of case samples. This paper highlights the issues found when working with hundreds of samples and shows a way to overcome them and succeed in the integration of the data from multiple batches.

## 2. Results

### 2.1. Instrumental Analysis Considerations

For this study, some analytical conditions were taken into account before analysis. Mobile phase volumes for the complete analysis in each mode were calculated beforehand, preparing 5 L of each, to avoid instrumental variability during the experiment. As the bottles for the mobile phases, needle and needle-seat cleaning and those for the extra pipelines of the pump did not fit on top of the HPLC, one of the 5 L bottles was randomly selected and placed on a separate stool. The rest were arranged on top of the equipment (see Figure S1). Samples were prepared in small sets (*n* = 32 per day) as the equipment required to analyze them as fresh as possible. Once the samples were prepared, they were kept on the autosampler tray until the end of the analysis. Between modes, samples were centrifuged before measurement to settle down any precipitate. The MS ionization source was cleaned between batches, but the chromatographic column was not, to avoid any potential column de-conditioning which could modify its chromatographic performance. The computer of the equipment was rebooted at the beginning and between the modes of analysis.

### 2.2. Sample Measurement

**Figure 1:**
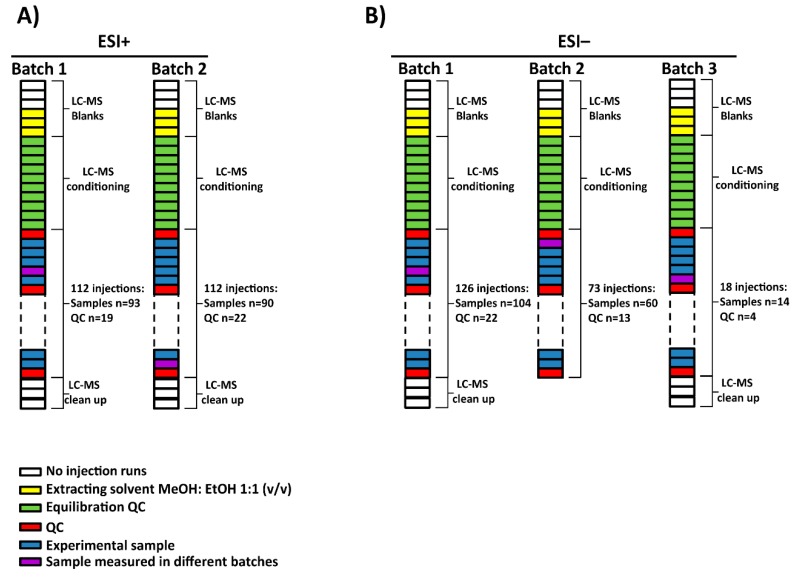
Experimental worklists and batches followed for (**A**) ESI+ and (**B**) ESI− modes. (**A**) Experimental samples were measured into two batches for ESI+ while (**B**) in the case of ESI− mode, 3 batches were obtained due to an ‘instrument communication error’. NOTE. The source of the equipment was not cleaned up between batches 2 and 3 in ESI− mode.

For this project, a total of 165 serum samples were measured in multiple batches. Two subsets of 7 samples among them were randomly selected to be analyzed in all batches in electrospray source ionization (ESI) in both positive (ESI+) and negative (ESI−) modes, respectively. The samples, blanks and QCs were analyzed following this worklist in each batch: first, 3 no-injection runs, 3 injections of extracting solvent (methanol: ethanol 1:1 (*v/v*)), 10 QC injections for system conditioning, one QC injection followed by 5 experimental samples until all were measured and finally, after the last QC injection, 3 no-injection runs. The no-injection runs were characterized by passing mobile phase through the column without injection in the port of the instrument. They were done with the aim of cleaning and starting the conditioning of the chromatographic conditions. Additionally, extracting solvent injections were used as blanks to identify and exclude signals coming from the experimental samples. The no-injection runs allowed the identification of signals from the column carry-over while the blank runs contained the injector carry-over as well. Regarding the ESI+, the analysis of the samples was done in two batches (see Figure 1A). On the other hand, in the case of ESI−, the samples were measured in three batches as the equipment stopped due to technical issues (an injection failure stopped the analysis) while measuring batch 2. As a result, batch 2 was subdivided into two: batch 2 and 3. Furthermore, batch 3 stopped due to an ‘instrument communication error’ that caused the worklist to fail, impeding sample acquisition. As a result, complementary samples were not measured, including a sample selected to be measured in both batches (see Figure 1B). To sum up, 183 and 178 experimental samples were measured in ESI+ and ESI− mode, respectively.

### 2.3. Analysis of the Labeled IS Mix

IS mix was used to monitor the performance of the system in LC-QTOF-MS experiments (7). For this study, the selection of the compounds for the IS was focused on different physicochemical properties including a lysophosphocholine (LPC), a sphingolipid, a fatty acid, an amino acid and a carnitine with a wide range of *m/z* values and retention times (RTs) (see Figure S2). Carnitine-D_3_ and sphingosine-D_7_ were detected only in ESI+ while stearic acid-D_5_ was only detected in ESI− mode. LPC18:1-D_7_—which showed two peaks at RT 19.3 and 20.0 min—and isoleucine ^13^C,^15^N were detected in both modes. 

Figure S3 depicts the variation in the relative IS intensities in the experimental samples and QCs as a function of the injection order, in order to evaluate the presence and composition of the IS mix. The IS mix was detected in all samples for both modes of analysis. Additionally, fluctuations in the abundances of the IS compounds were observed. Regarding the ESI+ mode, the most abundant compound was sphingosine-D_7_, while for ESI− mode it was LPC 18:1-D_7_—the peak at RT 20.00 min. 

**Figure 2:**
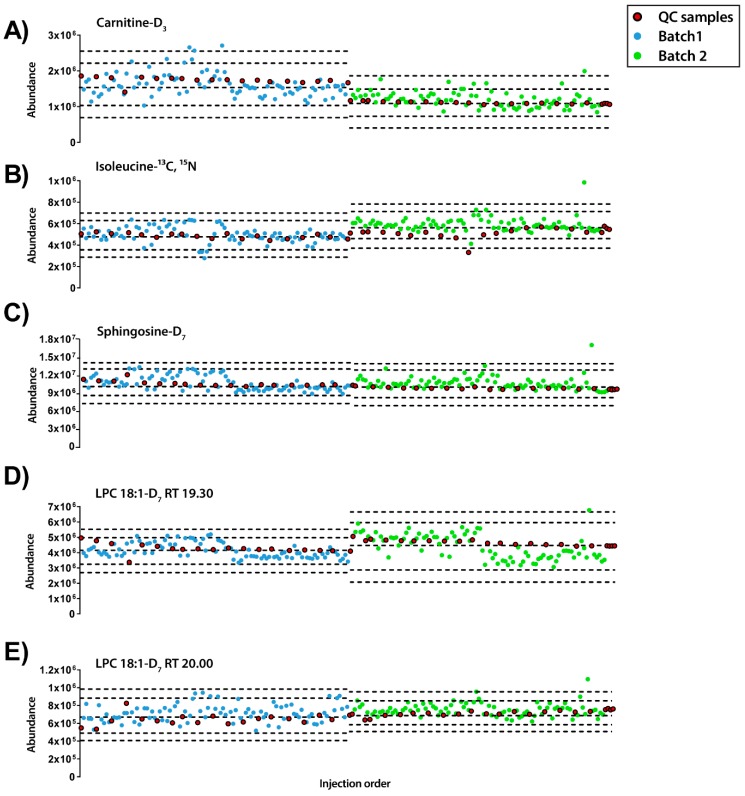
Quality control charts of the abundance of IS compounds in each sample according to the injection order for ESI+ mode. (**A**) Carnitine-D_3_ RT 0.70min, RSD = 22.95%. (**B**) Isoleucine-^13^C, ^15^N RT 0.77 min, RSD = 8.40%. (**C**) Sphingosine-D_7_ RT 14.38 min, RSD = 5.15%. (**D**) LPC18:1-D_7_ RT 19.30 min, RSD = 7.04%. (**E**) LPC18:1-D_7_ RT 20.00 min, RSD = 8.82%. **Legend**. *Y*-axis: Abundance. *X*-axis: Sample order. *Red circles*: QCs; *blue and green circles*: Experimental samples measured in batch 1 and batch 2, respectively. Dotted lines represent the mean +/− 2 and 3 SD for each batch independently.

**Figure 3:**
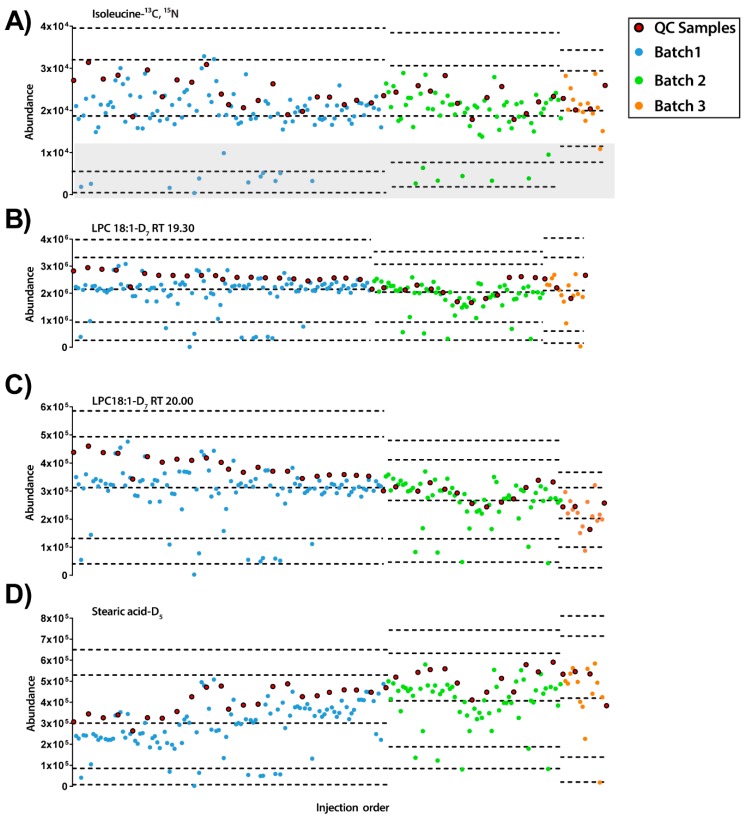
Quality control charts of the abundance of IS compounds in each sample according to the injection order for ESI− mode. (**A**) Isoleucine-^13^C, ^15^N at RT 0.77 min, RSD =25.01%. Grey rectangle signals samples with low levels of IS mix. (**B**) LPC18:1-D_7_ RT 19.30 min, RSD = 23.93%. (**C**) LPC18:1-D_7_ RT 20.00 min, RSD = 21.22%. (**D**) Stearic acid-D_5_ RT 34.54 min, RSD = 30.03% Legend. *Y*-axis: Abundance. *X*-axis: Sample order. *Red circles*: QCs; *blue and green circles*: experimental samples measured in batch 1 and batch 2, respectively. Dotted lines represent the mean +/− 2 and 3 SD for each batch independently.

Besides, the abundance of each IS compound was plotted along the injection order to observe their pattern in both modes of analysis (Figure 2 and Figure 3). Regarding the ESI+ mode, the plot showed that each IS had a distinct pattern within each sample (Figure 2). LPC 18-D_7_ can exemplify this, as it presented two peaks at 19.30 and 20.00 min and each of them followed a different pattern. Moreover, from the IS compounds that eluted at the beginning of the chromatogram, L-carnitine-D_3_ showed a trend to decrease the signal and such trend was not observed in isoleucine ^13^C, ^15^N. Finally, lower relative standard deviation (RSD%) values were observed in isoleucine- ^13^C, ^15^N, sphingosine-D_7_, LPC 18:1-D_7_ RTs 19.3 and 20.0 with RSD of 8.40%, 5.15%, 7.04% and 8.82%, respectively, compared to L-carnitine-D_3_ with a RSD of 22.95%.

On the other hand, in ESI− mode, it was detected that some of the experimental samples presented low abundance for all the IS compounds (see Figure 3). Furthermore, as observed previously with ESI+ mode, each IS compound had different inter- and intra-batch variability along the experiment. Interestingly, while stearic acid-D_5_ had a trend to increase its signal along the experiment, LPC18:1-D_7_ showed the opposite trend. Excluding the samples with low IS mix abundance (lower than the mean minus 2 standard deviation (SD), *n* = 20), the RSDs of the IS compounds were LPC 18:1-D_7_: 23.93% and 21.22% for 19.3 and 20.0 min, respectively, isoleucine-^13^C, ^15^N 25.01% and stearic acid-D_5_ 30.03%. 

To sum up, the use of IS with different physicochemical and biological properties has proved that each one may provide different information about the instrument performance and the presence of both intra- and inter-batch effects. Furthermore, results show that the information provided by the different IS is complementary, as the observed trends did not necessarily correlate with each other.

### 2.4. Initial Quality of the Data

Data was pre-processed by eliminating the signals from the blanks, filtering by presence on QCs, replacing missing values using the k-nearest neighbors (kNN) algorithm, and calculating the RSD% on QCs to exclude those LC-MS features showing RSD > 30%. As a result, the initial number of features for ESI+ and ESI− modes were 991 and 370, respectively. Therefore, a plot of total useful signal (TUS) following the injection order was performed to evaluate the response of each sample in the experiment Figure S4. We observed that the trend followed by the samples in ESI+ mode was similar to the ISs: LPC18:1-D_7_ at RT 20.00 min (see Figure 2D). Moreover, in the case of ESI− mode, an increasing signal from QCs along the experiment was observed. Interestingly, the experimental samples that showed low abundance of IS mix in ESI− mode did not show lower TUS signal.

Moreover, an unsupervised PCA model was built with the data excluding those samples with a TUS higher than 3 SD of the mean or lower than −3 SD to observe initial patterns of the samples (Figure S5). As we can observe in ESI+ mode the first component (PC1), which accounts for the 20.8% of the variability in the data set, separates both batches. There is a clustering of QCs within each batch. In the case of ESI− mode, the same pattern was observed with the exception that batch 2 and 3 were clustering together and that QCs showed a higher variation within each batch (Figure S6). In this case, PC1 and PC2 were investigated independently along with injection order (Figure S7). As it can be observed, batch separation was determined by the second component which accounted for 10.0% of total data variation while the variation in the first component, accounting for 35.9% of the total variance, was associated to the analytical drift. Finally, in ESI− the clustering of a group of samples in the left end of the plot (grey circle) matched with those samples with lower abundances of ISs. 

Together, the initial quality checking indicated that the normalization between batches is necessary before the start of the statistical analysis of the metabolomic profiles.

### 2.5. Normalization of Data

The strategy of normalization for data from different batches is critical, as there are no warranties that intra- and/or inter-batch effects will be accurately corrected afterwards. Two of the most common normalization approaches in metabolomics were initially applied: (1) Normalization by the sum of the IS mix compounds and (2) normalization by the TUS of each sample. These normalization approaches were evaluated using both a TUS plot represented according to the injection order and a principal component analysis (PCA) model (Figures S6 and S8). Regarding the normalization by IS mix, TUS plots for both modes showed a clear trend to increase the signal along the injection order. This correlates with the PCA plot which displayed an analytical drift on the first PC. Moreover, separation of batches was still observed in both modes. Therefore, it is clear that normalization by IS mix does not correct the analytical drift observed in the experiment. The normalization by TUS showed similar QC abundance levels for all batches. However, PCA models showed comparable results as the IS mix normalization in terms of analytical drift and batch separation.

**Figure 4:**
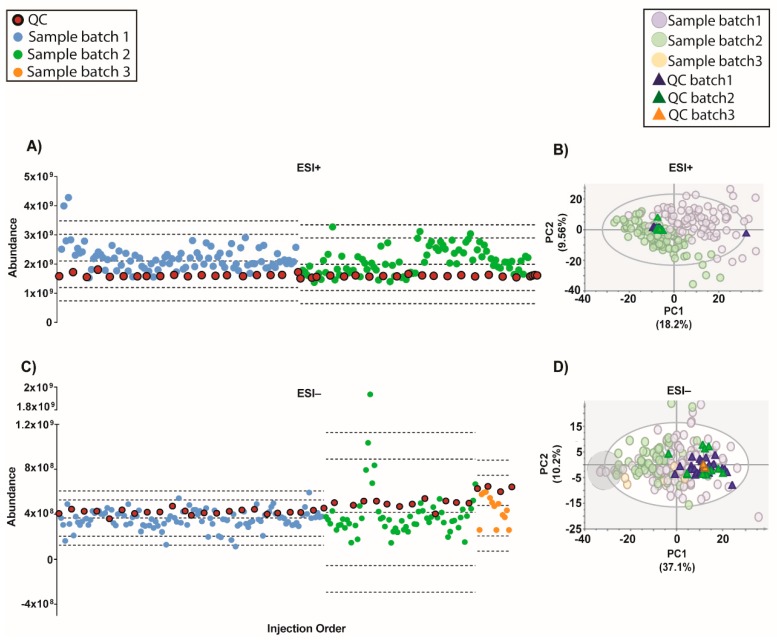
Outcome of the data normalization strategy after QC-SVRC algorithm for both ESI+ and ESI− modes. (**A**,**C**) Quality control chart of ESI+ and ESI− modes. Samples with a TUS higher than 3 SD of the mean or lower than −3 SD were removed from PCA model. (**B**,**D**) PCA plots of ESI+ and ESI− mode, respectively Signals with %RSD < 30% on QCs were kept, and UV scaling was used. Features in ESI+ and ESI− modes, respectively: 1056 and 394. **Legend**. *TUS plot*: Blue and green dots: samples of batch1 and batch2, respectively, red dots: QCs. Dotted lines represent the mean +/− 2 and 3 SD for each batch independently. *PCA*: Blue dots and dark blue triangles are samples and QCs measured in batch1, respectively; green dots and dark green triangles are samples and QCs from batch2, respectively. Grey circle are samples with low levels of IS mix.

An algorithm called QC-SVRC for intra-batch effect correction based on the information contained on QCs was recently developed (10). This method was applied to correct the intra-batch effect for both modes (Figure 4). As shown by Figure 4A, the QC draw a straight constant line between batches for both modes. Regarding the PCA models, the QC clustering between batches was observed Figure 4B.

**Figure 5:**
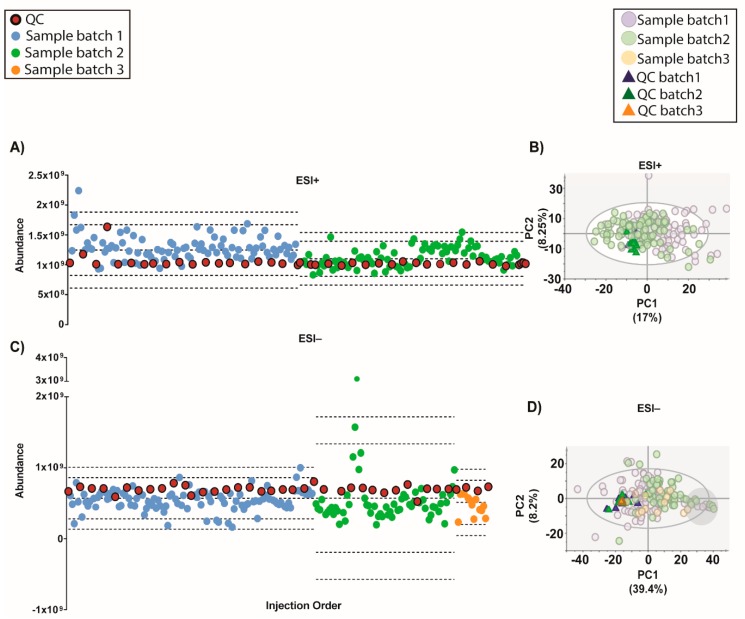
Outcome of the data normalization strategy after QC-SVRC algorithm and QC-norm for both: ESI+ and ESI− modes. (**A**,**C**) Quality control chart of ESI+ and ESI− modes, respectively. Samples with a TUS higher than 3 SD of the mean or lower than −3 SD were removed from the PCA model. (**B**,**D**) PCA plots of ESI+ and ESI− mode, respectively. Signals with %RSD < 30% on QCs were kept, and UV scaling was used. Features in ESI+ and ESI− modes respectively: 880 and 525. **Legend**. *TUS plot*: Blue and green dots: samples of batch1 and batch2, respectively, red dots: QCs. Dotted lines represent the mean +/− 2 and 3 SD for each batch independently. *PCA*: Blue dots and dark blue triangles are samples and QCs measured in batch1, respectively; green dots and dark green triangles are samples and QCs from batch2, respectively. Grey circle signals samples with low levels of IS mix.

Moreover, a recent strategy to normalize the inter-batch variation on top of QC-SVRC algorithm was used to obtain a complete integration of the data [4]. This method, called QC-norm, is based on the shift of QC median within and between batches [7]. The results are shown in Figure 5 and compared to the previous normalization we can observe the complete clustering of the QCs and the mix of the samples between batches. Results showed that a two-step combination of QC-SVRC algorithm followed by QC-norm enabled a successful correction of within and between systematic errors, thus facilitating the joint analysis of metabolic profiles acquired in independent batches.

Interestingly, the PCA model for ESI− raw data and after most normalization procedures (except by IS) showed a group of samples clustering together (marked within a grey circle). These samples presented a normal total ion chromatogram (TIC), TUS intensity values and clinical parameters comparable to the rest of the samples. However, as these samples presented low intensity of IS mix, they can be excluded from further statistics. Without the information of the IS mix, these samples could not have been excluded and would lead into the misinterpretation of the data.

**Figure 6:**
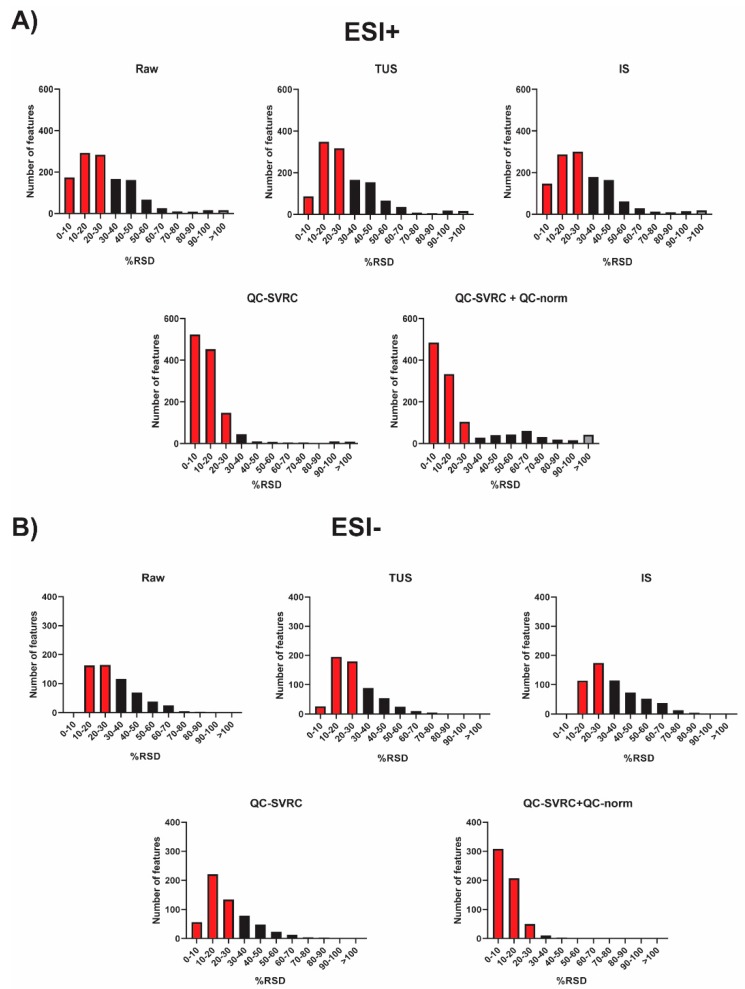
RSD distribution across QC samples from the complete dataset after each normalization method. (**A**) ESI+ mode and (**B**) ESI− mode. The red and grey bars indicate peaks that fall under RSD < 30% and RSD > 100%, respectively.

To sum up, all the normalization strategies tested were compared and the RSD% of all the features in QCs for both positive and negative ionization modes was plotted (Figure 6). As it can be observed, the combination of QC-SVRC +QC-norm showed the highest number of features with RSD < 30%. 

### 2.6. Assessment of Normalization Quality

In order to test the quality of the QC-SVRC normalization followed by QC-norm, four tests were used. First, PCA plots without QCs were evaluated in both modes for raw data (before normalization) and after normalization by QC-SVRC and by QC-SVRC + QC-norm (Figure S9). The latter procedure successfully integrated the samples measured in the multiple batches in both ionization modes. Moreover, in ESI− mode, the clustering of the samples with low IS mix abundance was still observed (grey circle).

Second, replicates of experimental samples were measured in different batches to assess the impact of the normalization strategy. This was done based on a PCA model again for raw data (before normalization) and after normalization, by QC-SVRC and QC-SVRC + QC-norm for both ionization modes (Figure S10). Once again, the intra- and inter-batch normalization procedure brought together the replicate samples in the PCA plots. Additionally, the distances of the repeated samples in these plots were calculated before and after normalization (QC-SVRC + QC-norm). The results were plotted and tested using a paired *t*-test (Figure S11). In the data from ESI+ mode, the difference in distances from the replicates was significantly lower (from 31.2 to 22.5) with a *p* value = 0.022 after normalization. Regarding the ESI− mode, the difference in distance was lower (from 12.8 to 9.1) but not significant (*p* value = 0.149).

**Figure 7:**
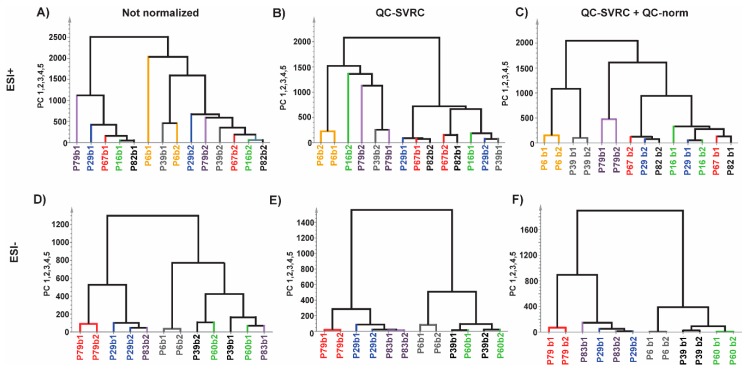
Normalization impact evaluated by HCA test on repeated experimental samples for (**A**,**D**) raw data; (**B**,**E**) after normalization by QC-SVRC and; (**C**,**F**) QC-SVRC + QC-norm for both, ESI+ and ESI− modes, respectively. **Legend**. Every pair of samples represents one patient and is depicted in a different color for each polarity mode.

Furthermore, hierarchical clustering analysis (HCA) was also performed to the same datasets (Figure 7). In ESI+ mode, no pairs of repeated samples were clustered before normalization, one pair clustered after QC-SVRC method and three pairs clustered after QC-SVRC+QC-norm. Regarding the ESI− mode, two pairs clustered in the HCA before normalization, three pairs after QC-SVRC and four after QC-SVRC+QC-norm. Moreover, the pairs that did not cluster together at the end of the normalization process were joined together in the proximal nodes of the tree (Figure 7C,F).

Third, the effects of the normalization in a metabolite were analyzed depicting its signal along the injection order before and after normalization (QC-SVRC followed by QC norm). Lysophosphocholine 20:0 (LPC 20:0, RT 29.50 min) was chosen as it was detected in both modes (Figure S12). Results in ESI+ mode showed minimal changes in the samples and QC, while in ESI− mode the correction of the signal from LPC 20:0 was observed. To conclude, these tests demonstrated that a normalization strategy can be easily implemented for the correction of systematic intra- and inter-batch effects. 

Finally, two clinical groups were selected and compared before and after normalization. Statistically significant features were compared using Venn diagrams (Figure S13). The total amount of significant features highly increased for both ionization modes.

## 3. Discussion

The execution of large-scale metabolomic studies is a complex task, specially using LC-QToF-MS, as the analysis of samples is typically distributed across different batches. The main issue is that differences in the instrument response across batches introduce a systematic error in the datasets that may lead to underpowered or even incorrect results in downstream analysis, leading ultimately to false discoveries. The aim of this study was to present a strategy to troubleshoot the integration of the data from different batches. 

In this study, analytical conditions for the large-scale experiment were described. We considered several aspects that should be taken into consideration before the analysis, such as the total mobile phase volume needed and their placement. Complete volume preparation will avoid changes in the ionization and chromatographic separation during the experiment. Additional factors we consider important and are mentioned in this study are: rebooting the computer before the experiment to ensure enough memory to complete the whole experiment, avoiding communication errors between computer and equipment; preparation of samples in subsets and centrifugation of sample vials before each analysis. Batches should also be designed to ensure that study groups are evenly distributed across batches. Accordingly, in this study, samples were split in two batches, and each batch included approximately the same number of cases and controls. 

Regarding QC preparation, this is not an obvious task. We considered that a quarter of the samples from each experimental group were necessary to obtain a representative sample of our experimental population (i.e., 46 samples in this study). Following this approach, the resultant QC was representative of the tested population and fulfilled the objective of evaluating the performance and stability of the analytical technique. Moreover, in order to avoid repeated freeze-thaw cycles, the selected experimental samples for the QC were assigned to be measured in the first batch and as the first set of samples, with the idea of preparing at the same time each experimental sample and QC. This QC was pooled into different tubes with the aim to thaw new QCs as needed. After the preparation of this first set of samples, we calculated the samples analyzed per day. As a consequence, we prepared a new set of samples each day to maintain a comparable time span to the first sample set.

Peak areas from the IS mix were assessed for a preliminary analysis of data quality after acquisition. Traditionally, the main use of a IS in metabolic fingerprinting in other separation techniques (such as such as gas chromatography and capillary electrophoresis) has been to correct the volume of injection and the MS signal of each sample [14,15,16]. In the case of LC-QToF-MS, especially in large-scale experiments, IS are useful for the monitoring of performance of the system [7]. In this study, a combination of 5 ISs was employed: L-carnitine-D_3_, isoleucine-^13^C, ^15^N, sphingosine-D_7_, LPC 18:1-D_7_ and stearic acid-D_5_. The aim of this IS mix was to cover not only the complete RTs of the chromatogram (from 0.6 to 34.5 min) but also a wide spectrum of biochemical and physicochemical properties. The results markedly showed that each signal from the IS mix followed a different trend for the same sample in both ionization modes. Moreover, these independent trends did not correlate with compound abundances, the RT in the chromatogram or the nature of the IS. This fact suggests the idea that each biological sample has an intrinsic matrix effect.

In case of the IS mix in ESI− mode, we found that some samples presented very low intensities of all IS peaks. These samples were not present together in a specific time point of the experiment but were throughout all the batches. This issue is intriguing since it might suggest technical issues in metabolite extraction, but no differences were observed on the TUS values of these samples. Additional experiments are needed to understand this effect.

Moving forward, the quality of the initial data (after filtration of blanks, missing values and %RSD in QCs) showed that although on the TUS were comparable among batches, they were different indeed in the PCA plots (Figure 4 and Figure S4). This evidence proves that a normalization strategy is necessary to integrate the data from the whole experiment.

In the case of data normalization, we tried two common strategies applied in metabolomics: the normalization by IS (in this case the sum of all IS compounds) and the normalization by TUS. The results show that both approaches did not achieve the integration of the data and, in the case of TUS normalization, increased the drift of the QC by the injection order.

However, in the latest years the normalization methods based on the information contained on QCs has gain great interest to correct the instrumental drift, especially in large-scale studies [10,11,12]. Most of these, have showed the application of support vector regression (SVR) for the modeling of the instrumental drift, such as the strategy proposed by Kuligowski et al. in 2015 [10], which uses a radial basis function (RBF) kernel to correct the instrumental drift. This algorithm applied to our data, resulted in a good clustering of QCs from all batches in both modes. Moreover, following Sanchéz- Illana et al. in 2018 an inter-batch correction was applied [4]. The resulting outcome improved the complete integration of QCs in both modes (Figure 6). The samples with low IS mix in ESI− mode grouped together in one end of most PCAs (Figure 3, Figure 4, Figure 5, Figure 6 and Figure 7, Figures S5–S7 on grey circles) from the different normalization strategies. This demonstrates the importance of adding multiple IS to the samples. Otherwise, it could be thought that these samples shared a common biological condition.

The assessment of the QC-SVRC normalization followed by QC-norm, compared to raw data and with QC-SVRC alone, was tested by the analysis of the PCA models of complete set of samples and the comparison of samples measured in multiple batches (Figures S10 and S11, respectively). This last strategy has not been explored previously and proves the feasibility of the normalization strategy. After testing the reduction in distance of the replicates by the normalization we corroborate these findings by HCA where the clustered pairs increased after every normalization (intra and inter) for both modes (Figure 7). Additionally, the extent of normalization was checked by exploring the outcome in one of the metabolites common to both modes. We detected that the normalization process only corrects the effects that are necessary as in ESI+ mode there was no visual change compared to ESI− mode. To sum up, these tests demonstrate the full integration and the high quality of the data from multiple batches. This would result in the further reliable comparison of the clinical groups of the study. This was depicted by the significant number of features increased after normalization between two clinical groups, which otherwise would lead to false negative results.

To conclude, this work illustrates a strategy to generate high-quality data from untargeted large-scale metabolomics studies. This strategy considers the stability of the equipment and the normalization of the data to assess a correct integration of the batches. We have addressed different issues that should be taken into consideration in these types of studies. Together, we have generated a guideline to integrate data for different batches that could be useful in any metabolomics study with more than a single batch.

## 4. Materials and Methods 

### 4.1. Patients and Sample Collection

140 adult patients with a clinical history of asthma, and 25 non-allergic and non-asthmatic subjects were enrolled as control population at the allergy department, “Hospital Universitario de Gran Canaria, Dr. Negrin”, Las Palmas de G.C., Spain. Participants were recruited as they arrived to daily practice. All participants read and signed an informed consent. The study was conducted in accordance with the Declaration of Helsinki, and the protocol was approved by the Ethics Committee of “Hospital Universitario de Gran Canaria, Dr. Negrin” on the 4th of February 2016 (code: 160009).

Whole blood was collected and incubated with a clotting agent in Vacutainer™ SST II tube (Becton Dickinson S.A., Spain). The sample was placed at room temperature for 40 min. Afterwards, it was centrifuged at 2000 × *g* for 10 min at room T. Then, serum was collected and stored at −80 ℃ until the metabolomic analysis was performed.

### 4.2. Sample Randomization 

Due to the large number of samples, the sample set was split into 2 batches according to clinical parameters to obtain half of each experimental group in each batch. Thus, 83 and 82 samples were included in batches 1 and 2, respectively. In addition, to assess the correction of systematic between-batch effects, a subset of randomly selected samples was measured in both batches (7 and 6 samples in ESI+ and ESI− modes). Samples within batch were randomized for sample preparation and instrumental analysis.

### 4.3. Metabolomic Analysis

Serum samples were measured using an Agilent HPLC system (1200 series) coupled with quadrupole-time of flight analyzer system (Q-ToF MS 6520) (Agilent Technologies, Waldbronn, Germany), as previously described [17,18].

### 4.4. Sample Treatment

Serum proteins were removed adding 300 µL of cold (−20 °C) methanol: ethanol (1:1) to 100 µL of sample. For the analysis, 20 µL of a mix of five labelled IS were added to each sample. The IS mix was selected to cover a wide range of different RTs and physico-chemical properties. This was composed of: (1) Carnitine-D_3_ (C_7_H_12_D_3_NO_3_, mass = 144.1454 Da, RT: 0.70 min, 0.02 mM); (2) Isoleucine-^13^C, ^15^N (^13^C_6_H_13_^15^NO_2_, molecular mass=138.1118 Da, RT: 0.77 min, 0.18 mM); (3) Sphingosine-D_7_ (C_18_H_30_D_7_NO_2_, molecular mass = 306.3264 Da, RT: 14.38 min, 0.02 mM); (4) LPC 18:1-D_7_ (C_26_H_45_D_7_NO_7_P, molecular mass = 528.3921 Da, 2 peaks at RTs = 19.30 and 20.00 min, 0.01 mM) and (5) Stearic acid-D_5_ (C_18_H_31_D_5_O_2_, molecular mass = 289.3029 Da, RT: 34.54 min, 0.17 mM). All detected adducts are present in Table S1. Samples were then vortex-mixed and kept on ice for 5 min. Supernatant was separated by centrifugation (16110 × *g* for 20 min at 4 °C) and transferred into LC vials for analysis. Extraction solvent used as blank for the analysis was prepared mixing 400 µL of cold (−20 °C) methanol: ethanol (1:1) and 20 µl of ISs. The blank followed the same steps as the samples. 

### 4.5. QC Preparation 

QC was prepared by pooling equal volumes of serum from the first 46 samples of the first batch. Then, the QC was aliquoted into different tubes to preserve it from freezing-thawing cycles. Every time QC was needed, fresh QC was prepared. The QCs clean-up steps followed the same procedure applied for the experimental samples. QCs were analyzed throughout the run to provide a measurement of system stability, performance and reproducibility of the LC-QToF-MS system. 

### 4.6. LC-Quadrupole Time of Flight-MS Analysis

The HPLC system was equipped with a degasser, two binary pumps, and a thermostated autosampler. Briefly, 10 μL of sample were injected into a Discovery HS C18 column (2.1 × 150 mm, 3.0 μm; Supelco, Sigma Aldrich, Germany), with a guard column Discovery^®^ HS C18 (2.1 × 20 mm, 3 μm; Supelco), both maintained at 40 °C. The flow rate was set at 0.6 mL/min. The elution gradient involved a mobile phase consisting of: (A) 0.1% *v/v* formic acid (FA) in water and (B) 0.1% *v/v* FA in acetonitrile. The initial conditions were set at 25% phase B, which increased linearly to 95% phase B in 35 min. Then it returned to the initial conditions in 1 min, which were held for 9 min for column reconditioning. Samples were analyzed in both ESI+ and ESI− modes in separate injections. The capillary voltage was set at 3500 for ESI+ and 4000V for ESI−. The drying gas flow rate was 10.5 L/min at 330 °C and gas nebulizer at 52 psi; fragmentor voltage was 175 V; skimmer and octopole radio frequency voltages were set to 65 and 750 V, respectively. MS spectra were collected in the centroid mode at a scan rate of 1.2 Hz. The MS detection window was performed in full scan from 100 to 1200 *m/z* for both modes. Automatic MS recalibration during batch analysis was carried out introducing a reference standard into the source via a reference sprayer valve. Reference masses for ESI+ were purine (*m/z* = 121.0508) and HP-0921 (*m/z =* 922.0097), whereas for ESI− TFA NH4 (*m/z =* 119.0363) and HP-0921 (*m/z* = 966.0007). 

### 4.7. Raw Data Availability

Metabolomics data have been deposited to the EMBL-EBI MetaboLights database (DOI: 10.1093/nar/gks1004. PubMed PMID: 23109552) with the identifier MTBLS1133. The complete dataset can be accessed here https://www.ebi.ac.uk/metabolights/MTBLS1133 [19].

### 4.8. Data Treatment 

Acquired signals were processed to provide structured raw data in an appropriate format for analysis. Collected data from all batches were put and cleaned together in a single analysis of background and unrelated ions using MassHunter Profinder (B.08.00; SP3, Agilent Technologies) software. Molecular feature extraction (MFE) algorithm was used to reduce the size and complexity of data. Furthermore, Find by Ion (FbI) algorithm was applied to improve the reliability of the features found in the data. In the end, 1382 and 887 chemical signals for LC-MS positive and negative ionization modes were obtained respectively. Afterwards, raw data was filtered by keeping all features that were not present in the blanks, were detected in >50% of all QCs and >75% in the experimental samples. The rest of the signals were excluded from the analyses. Missing values were replaced using the kNN algorithm using an in-house script developed in Matlab^®^ [20].

The IS compounds were extracted in a targeted manner using a file containing the formula, mass and RT in MassHunter Profinder.

### 4.9. Data Normalization and Analysis

Different normalization strategies were used to pre-process the raw data matrix containing data acquired from the independent batches. For intra-batch normalization, the following methods were used: (1) Normalization of each signal (i.e., LC-MS feature) to the total area of the IS mix; (2) normalization of each signal by the TUS of each sample; (3) intra-batch effect correction using the QC-SVRC (10) algorithm using the information contained in the QCs to correct the drift of the MS signal within the batch. This algorithm requires the selection of the tolerance threshold (ε), the kernel width (γ) and the error penalty parameter (C). The values used were ε = 5%, γ = [2^−3^, 2^−2^, …, 2^6^], and C = 50%. In addition to intra-batch normalization using QC-SVRC, a sequential inter-batch effect correction called QC-norm was tested. It is a ratio-based method which normalizes the intensity of each signal in each sample using as scaling factor the ratio of the median intensity in the QC of the corresponding batch over the median QC intensity across batches (4). Moreover, after each normalization, features were filtered by keeping those with a RSD < 30% in QCs.

To monitor the performance of each data pre-processing strategy, the abundance of TUS as a function of the injection order was depicted before and after each normalization approach. In each of them, the mean plus/minus 2 and 3 times the SD were calculated and depicted for each batch independently.

Multivariate and HCA were performed using SIMCA^®^ v.15.0 (Umetrics, Umeå, Sweden). PCA, a non-supervised model, was used to identify patterns across samples associated to the injection order and batch after the data normalization. Autoscaling was used for all the models. HCA was performed for the samples analyzed in all batches to assess the similarity after normalization using Ward’s method to calculate the similarity between clusters. The trees were sorted by size. 

The distances in the PCA models of the repeated experimental samples were calculated and compared before and after normalization (QC-SVRC followed by QC-norm). Afterwards, using a paired t-student test the distances were tested. Moreover, the reduction of the distance between the pair of repeated samples after normalization was plotted calculating the Euclidean distance of the pairs in Matlab^®^.

To prove the relevance of data normalization, two clinical groups were compared before (using raw data) and after the normalization using QC-SVRC followed by QC-norm. The number of samples of each group were 9 and 17, and the applied statistical test was non-parametric Mann–Whitney U test using an in-house script developed in Matlab^®^.

## Supplementary Materials

The following are available online at https://www.mdpi.com/2218-1989/9/11/247/s1, Figure S1: Scheme of the mobile phase position during large-scale experiment, Figure S2: Extracted ion chromatogram (EIC) of IS mix in A) ESI+ and B) ESI− modes, Figure S3: Relative abundance of the IS mix in all experimental samples and QCs according with the injection order for ESI+ and ESI− modes, Figure S4: Quality control charts of the total useful signal (TUS) using the initial data (before normalization) for each sample according to the injection order, Figure S5: PCA models of initial data (before normalization), Figure S6: Outcome of the data normalization strategy by the IS mix for both: ESI+ and ESI− modes, Figure S7: Contribution of PC1 and PC 2 along with injection order in ESI− mode after normalization by IS mix, **Figure S8**: Outcome of the data normalization strategy by the TUS for both: ESI+ and ESI− modes, Figure S9: Normalization impact evaluated by PCA models of all experimental samples in the study, Figure S10: Normalization assessment by PCA of repeated experimental samples for raw data; after normalization by QC-SVRC and QC-SVRC + QC-norm., Figure S11: Euclidean distances plots of repeated experimental samples, Figure S12: Abundance plots before and after normalization of LPC 20:0, RT 29.5 min, Figure S13: Venn diagram comparing the number of significant features between two groups of the clinical study before normalization (raw data) and after QC-SVRC + QC-norm normalization., Table S1: IS mix adducts detected in the experiment.
