# Supplementary material for: Troubleshooting in Large-Scale LC-ToF-MS Metabolomics Analysis: Solving Complex Issues in Big Cohorts
Source: Metabolites. 2019 Oct 24;9(11):247. doi: 10.3390/metabo9110247 (PMC6918290; doi:10.3390/metabo9110247)
Supplement: Supplementary file 1 [file metabolites-09-00247-s001.pdf]

metabolites MDPI

Troubleshooting in large-scale LC-ToF-MS metabolomics analysis: solving complex issues in big cohorts

SUPPLEMENTARY MATERIAL

Figures

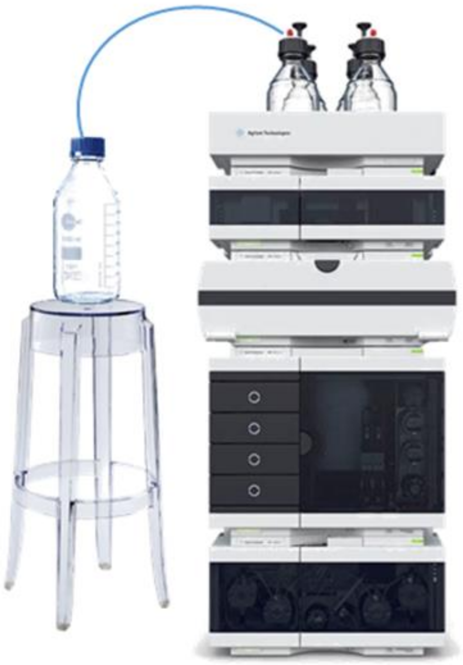

Figure S1. Scheme of the mobile phase position during large-scale experiment. In order to avoid variability in the mobile phases, 5 L of each (aqueous and organic phases) were prepared. As both mobile phases bottles did not fit on the top of the equipment, one was placed on a separate stool.

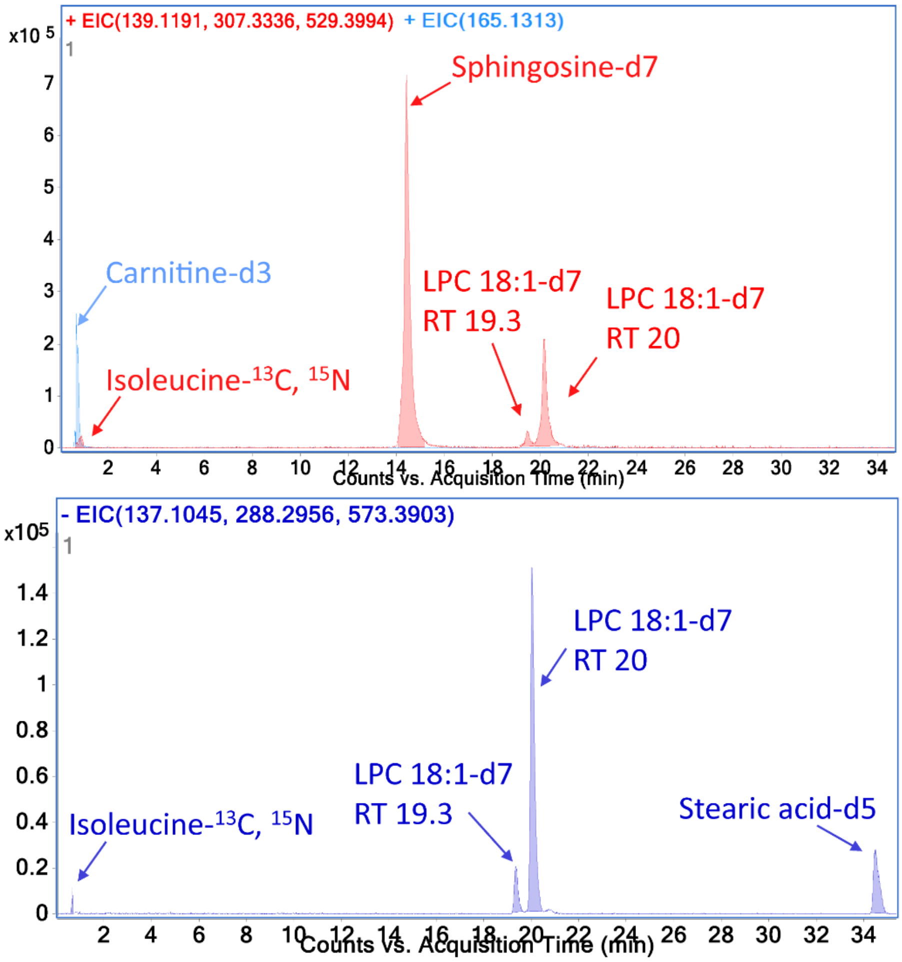

Figure S2. Extracted ion chromatogram (EIC) of IS mix in (A) ESI+ and (B) ESI- modes. (A) Detection from left to right: In blue: Carnitine-D_3 (*m/z* = 165.1313, RT = 0.70 min, adduct = $[M+H]^+$); in red: Isoleucine-^{13}C, ^{15}N (*m/z* = 139.1191, RT = 0.77 min, adduct = $[M+H]^+$), Sphingosine-D_7 (*m/z* = 307.3336, RT = 14.38 min, adduct = $[M+H]^+$) and LPC 18:1-D_7 (*m/z* = 529.3994, RT = 19.30 and 20.00 min, adduct = $[M+H]^+$). (B) EIC of IS standards measured in ESI- mode. From left to right in order of appearance: Isoleucine-^{13}C, ^{15}N (*m/z* = 137.1045, RT = 0.77 min, adduct = $[M-H]^-$), LPC 18:1-D_7 (*m/z* = 573.3903, RT = 19.35 and 20.00 min, adduct = $[M+COO]^-$) and stearic acid-D_5 (*m/z* = 288.2956, RT = 34.54 min, adduct = $[M-H]^-$).

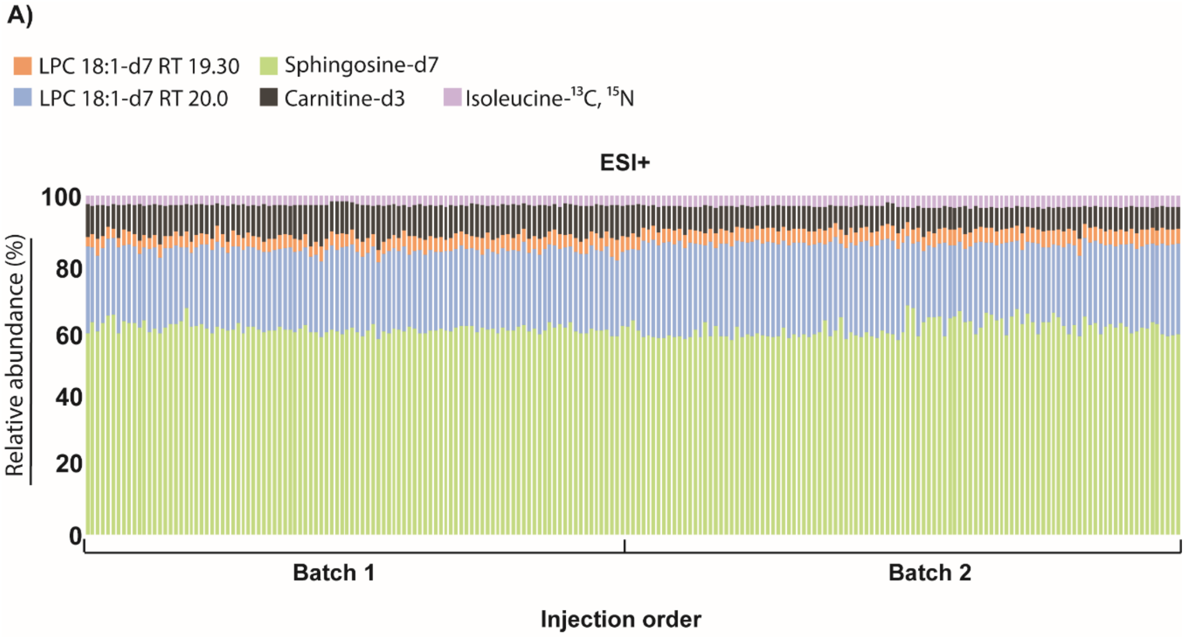

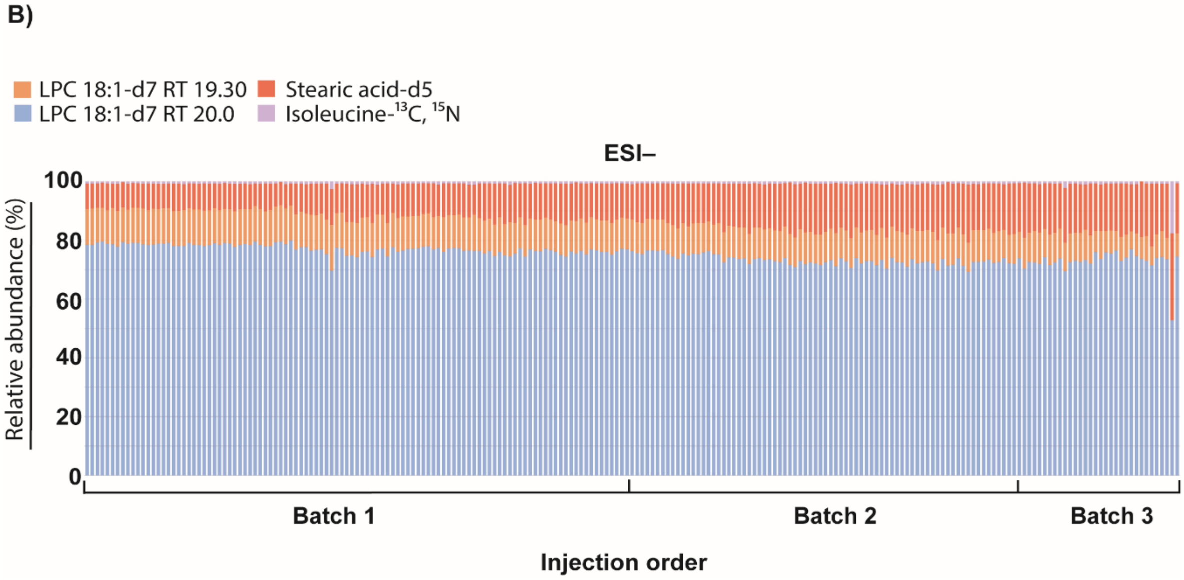

Figure S3. Relative abundance of the IS mix in all experimental samples and QCs according with the injection order for (A) ESI+ and (B) ESI− modes, respectively. Legend. *Black*: L-carnitine-d5; green: sphingosine-D_7, *orange*: LPC18:1-D_7, *red*: stearic acid-D_5 and *purple*: isoleucine- ^{13}C, ^{15}N.

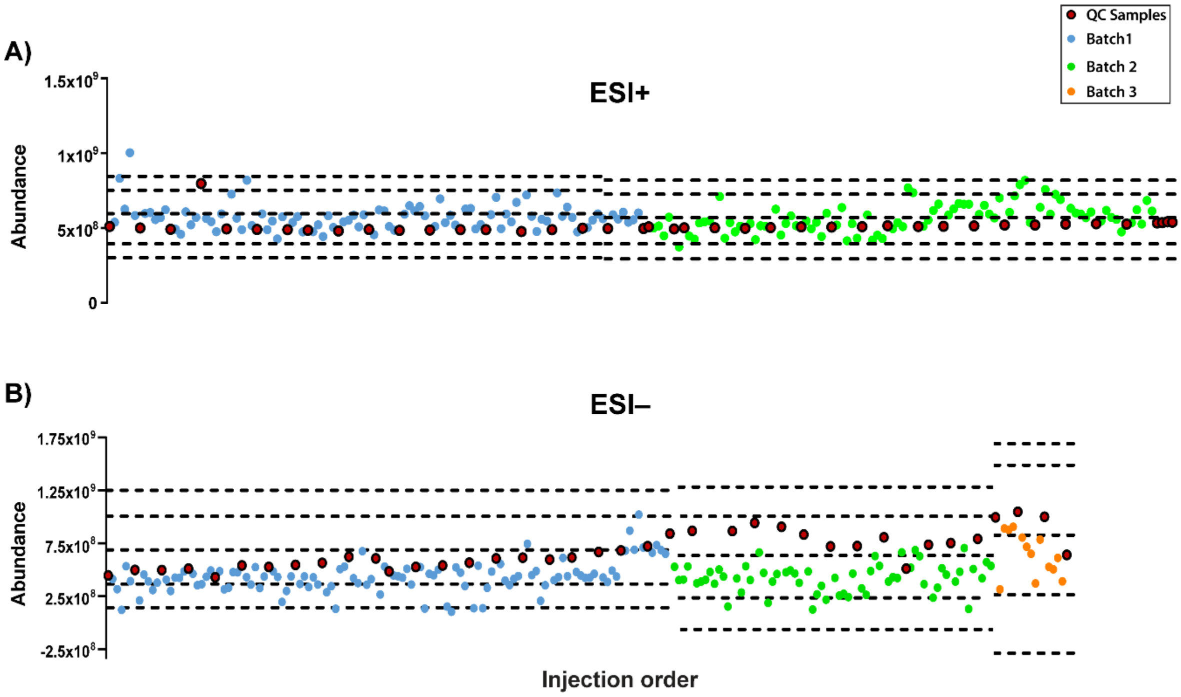

Figure S4. Quality control charts of the total useful signal (TUS) using the initial data (before normalization) for each sample according to the injection order. (A) ESI+ mode (features n = 991) (B) ESI− mode (features n = 370). Legend. *Y-axis*: Abundance. *X-axis*: Injection order. *Red circles*: QCs; *blue and green circles*: experimental samples measured in batch 1 and batch 2, respectively. Dotted lines represent the mean +/− 2 and 3 SD for each batch independently.

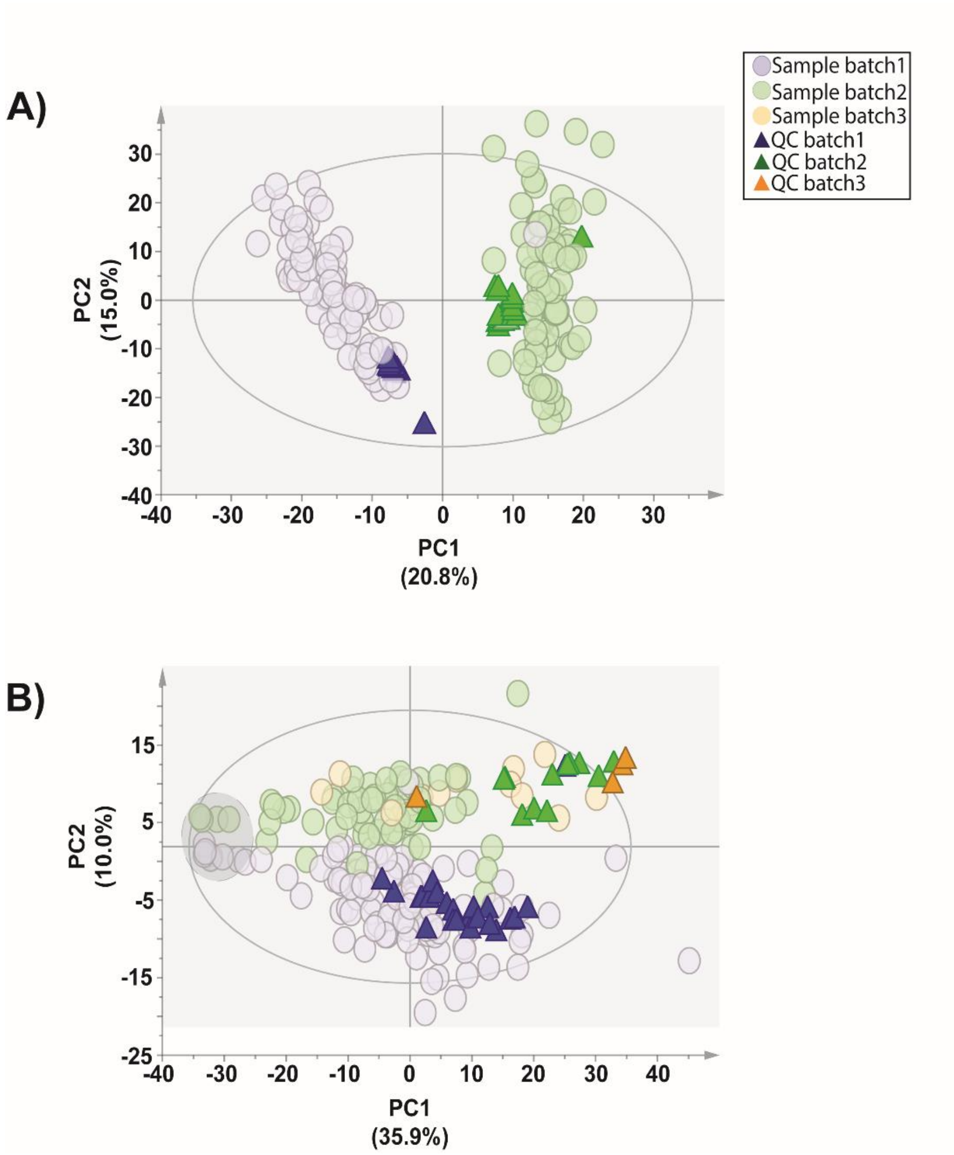

Figure S5. PCA models of initial data (before normalization). (A) ESI+ mode (features *n* = 991); (**B)** ESI− mode (features *n* = 370). Grey circle: samples with low levels of the IS mix. **Legend.** *Blue dots and dark blue triangles*: samples and QCs measured in batch 1; *green dots and dark green triangles*: samples and QCs from batch 2**.**

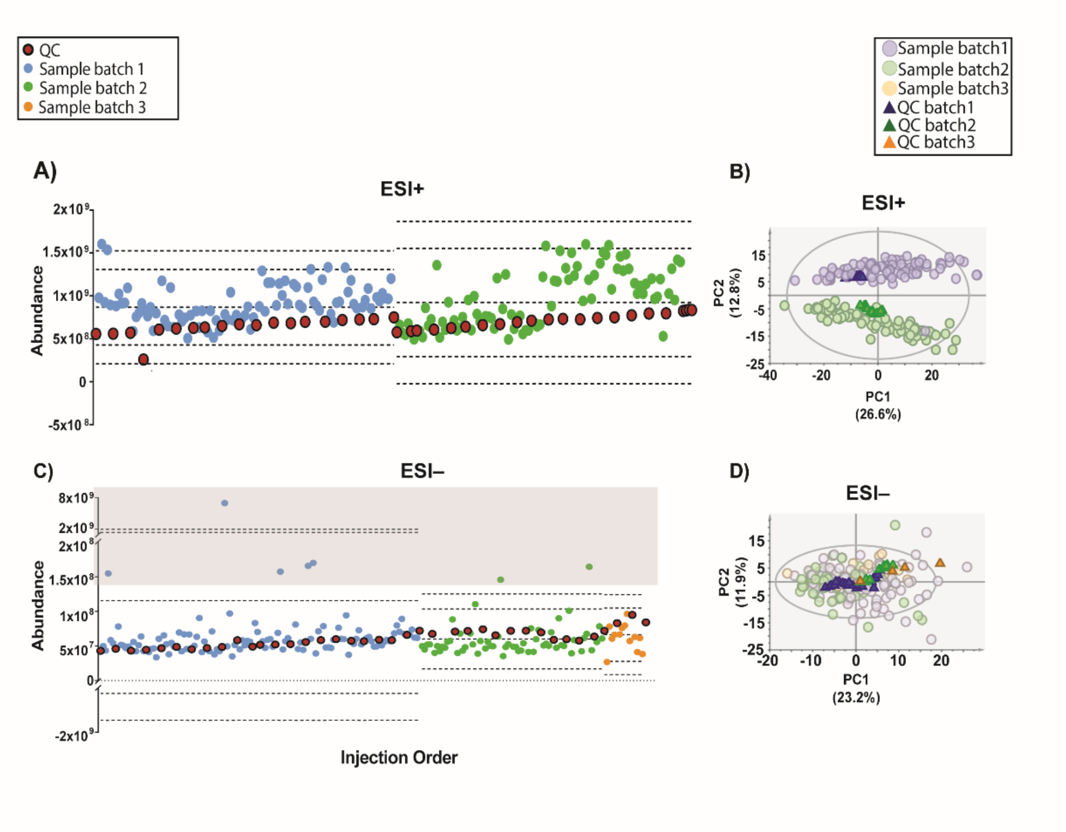

Figure S6. Outcome of the data normalization strategy by the IS mix for both: ESI+ and ESI− modes. (A,C) Quality control chart of the total useful signal (TUS) from ESI+ and ESI− modes, respectively. Grey rectangle shows the samples with low levels of the IS mix. Samples with a TUS higher than 3 SD of the mean or lower than −3 SD were removed from the PCA model. Features with %RSD < 30% on QCs were kept; ESI + mode (n = 698) and ESI- mode (n = 246). **Legend.** *TUS plot*: Blue and green dots: samples of batch1 and batch2, respectively, red dots: QCs. Dotted lines represent the mean +/− 2 and 3 SD for each batch independently. *PCA*: Blue dots and dark blue triangles are samples and QCs measured in batch 1, respectively; green dots and dark green triangles are samples and QCs from batch 2, respectively.

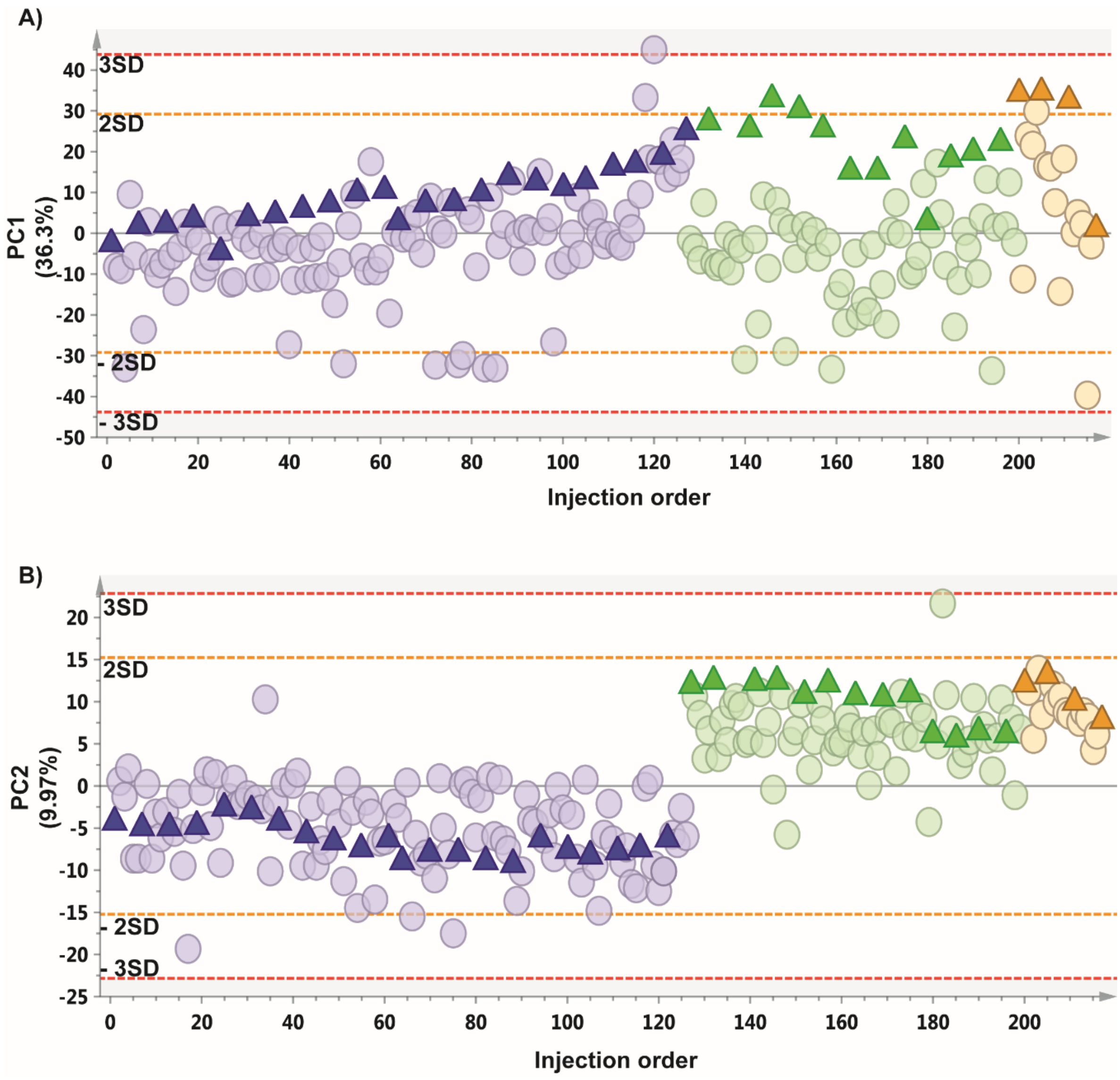

Figure S7. Contribution of (A) PC1 and (B) PC 2 along with injection order in ESI− mode after normalization by IS mix. In Y axis the variation of PC1 or 2. In X axis the injection order of the samples.

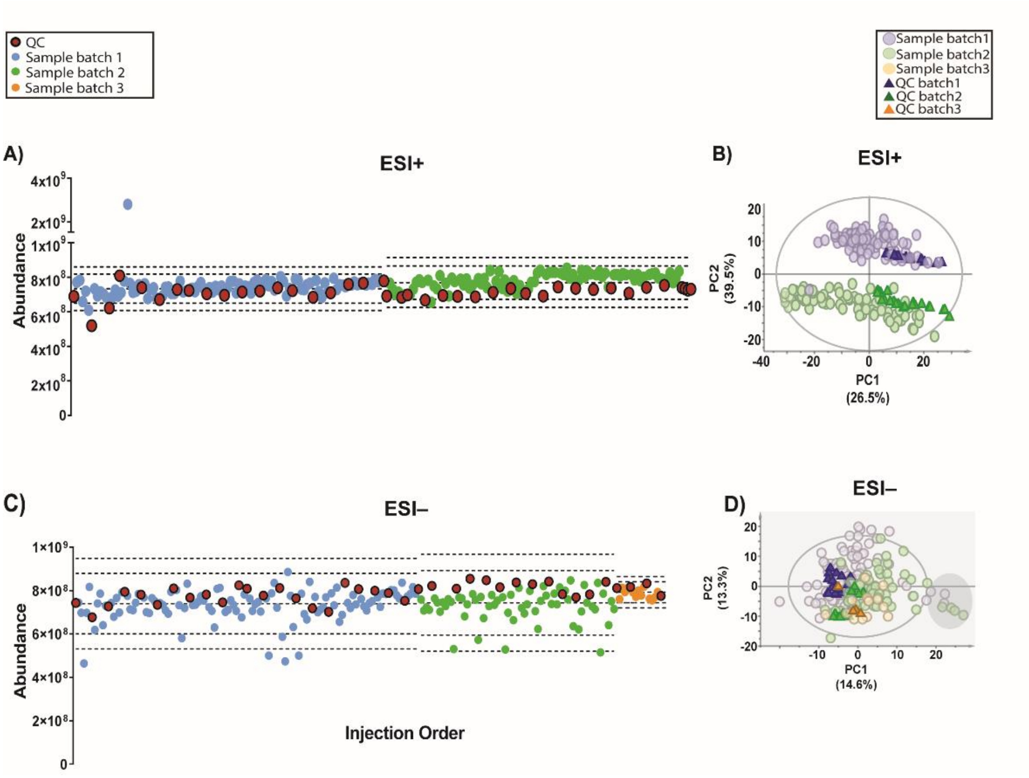

Figure S8. Outcome of the data normalization strategy by the TUS for both: ESI+ and ESI− modes. (A,C) Quality control chart of the total useful signal (TUS) from ESI+ and ESI− modes, respectively. (**B,D)** PCA plots of ESI+ and ESI- mode, respectively. Grey rectangle and circle, shows the samples with low levels of the IS mix. Samples with a TUS higher than 3 SD of the mean or lower than −3 SD were removed from the PCA model. Features with %RSD < 30% on QCs were kept; ESI + mode (n = 705) and ESI- mode (n = 356). **Legend.** *TUS plot*: Blue and green dots: samples of batch1 and batch2, respectively, red dots: QCs. Dotted lines represent the mean +/− 2 and 3 SD for each batch independently. *PCA*: Blue dots and dark blue triangles are samples and QCs measured in batch 1, respectively; green dots and dark green triangles are samples and QCs from batch 2, respectively.

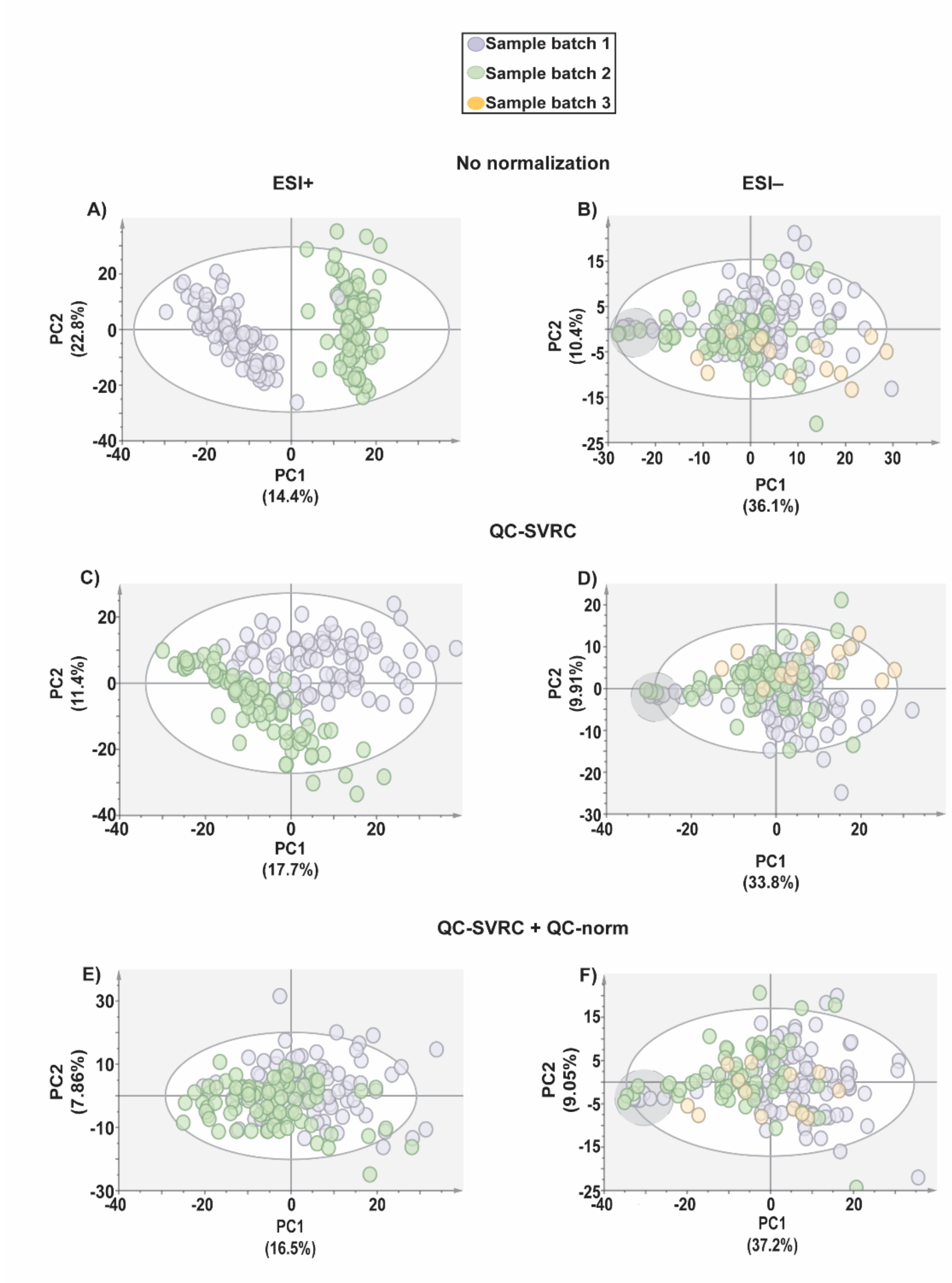

Figure S9. Normalization impact evaluated by PCA models of all experimental samples in the study for (A,D) raw data; (B,E) after normalization by QC-SVRC and; (C,F) QC-SVRC + QC-norm for both, ESI+ and ESI− modes, respectively. Legend. UV scaling was used. The blue, green and orange circles are samples measured in batch 1, 2 and 3, respectively. Gray circle signals samples with low levels of IS mix.

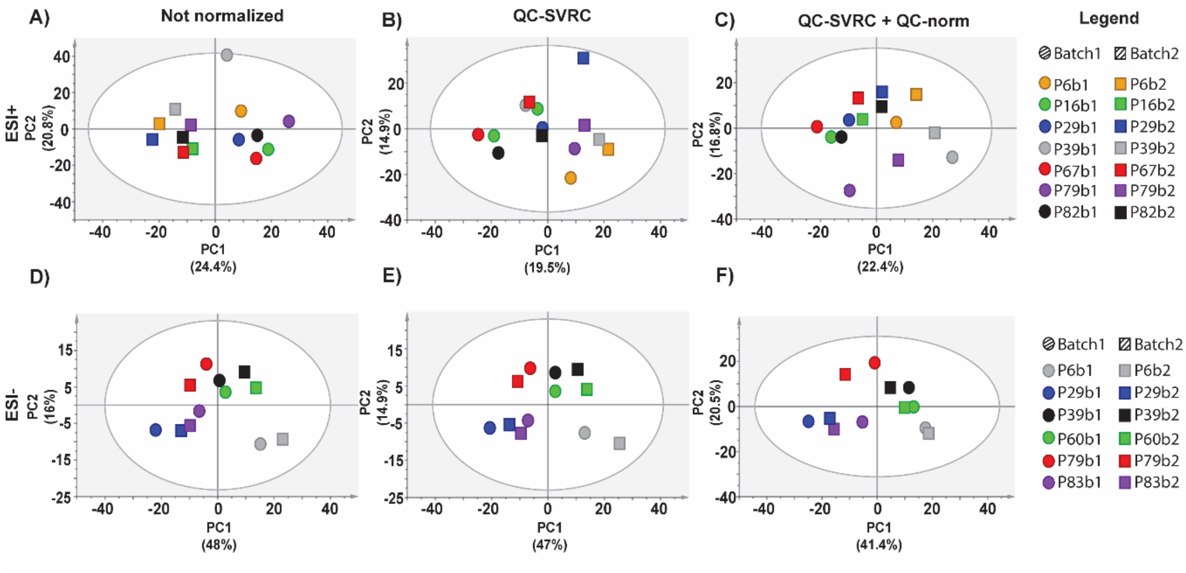

Figure S10. Normalization assessment by PCA of repeated experimental samples for (A,D) raw data; (B,E) after normalization by QC-SVRC and; (C,F) QC-SVRC + QC-norm for both, ESI+ and ESI− modes, respectively. Legend. UV scaling was used. Samples measured in batch 1 were depicted by a circle symbol while samples in batch 2 were represented by a square symbol. Every pair of samples represents one patient and is depicted in a different color for each polarity mode.

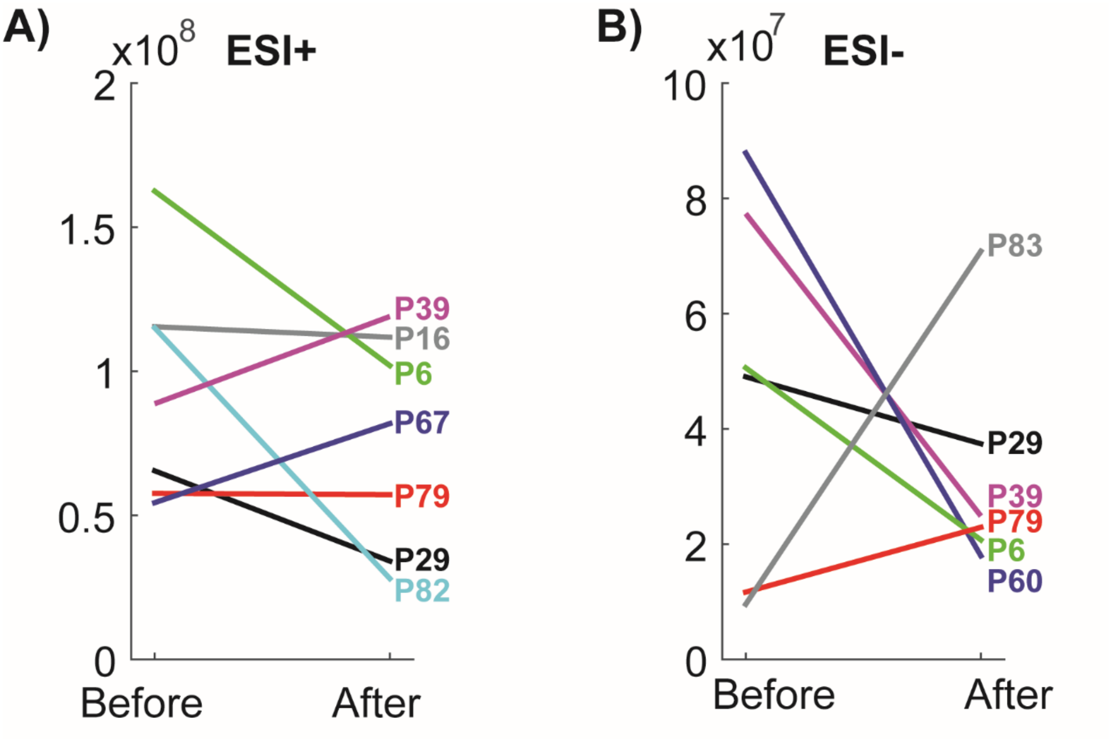

Figure S11. Euclidean distances plots of repeated experimental samples for both, (A) ESI+ and (B) ESI− modes, respectively. Legend. Every pair of samples represents one patient and is depicted in a different color for each polarity mode.

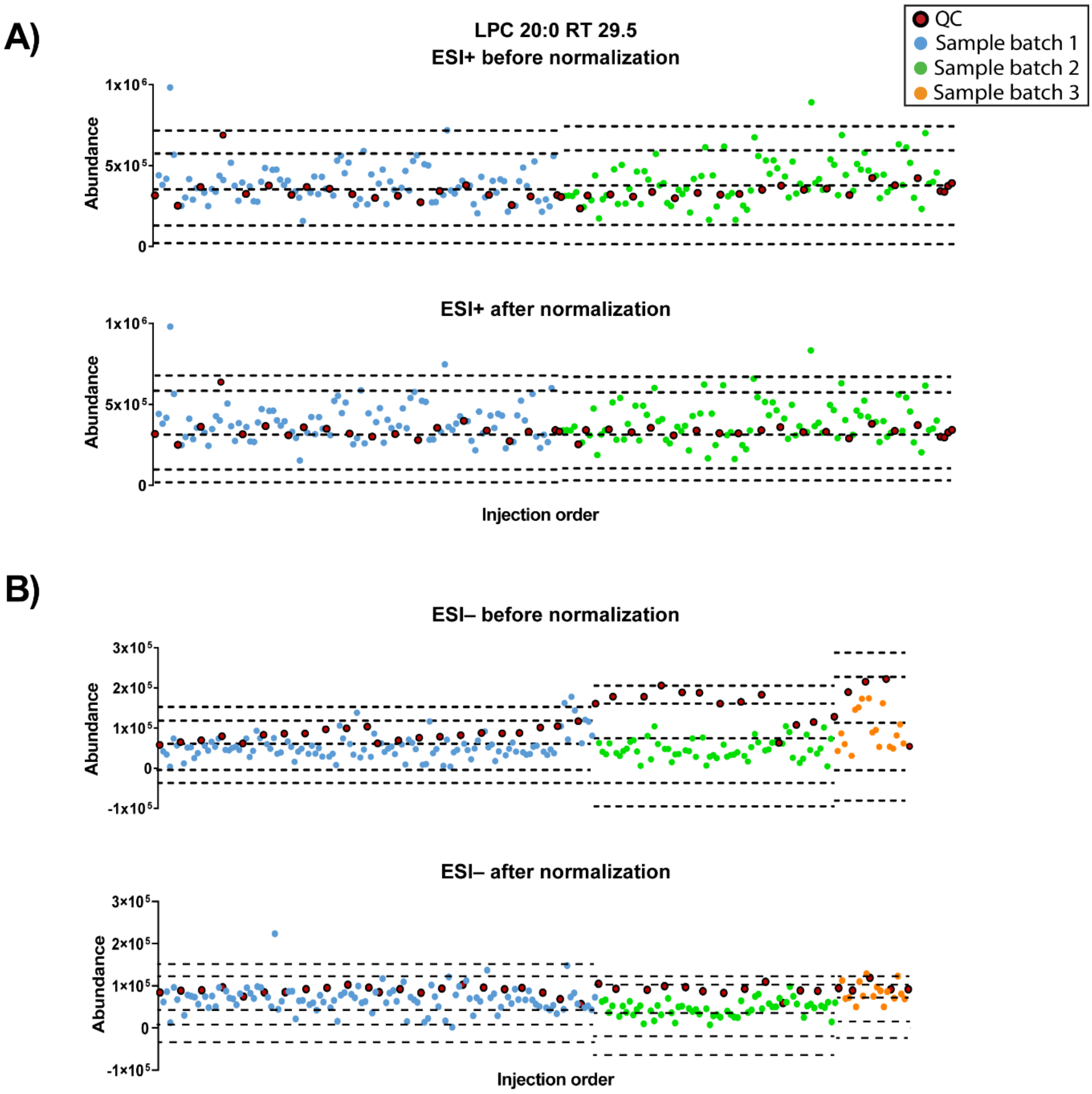

Figure S12. Abundance plots before and after normalization of LPC 20:0, RT 29.5 min in (A) ESI+ and (B) ESI− modes, respectively. Legend: Top; plot before normalization (QC-SVRC + QC-norm normalizations); bottom: after normalization; *red circles*: QCs; *blue, green and orange circles*: samples measured in batch 1, 2 and 3, respectively.

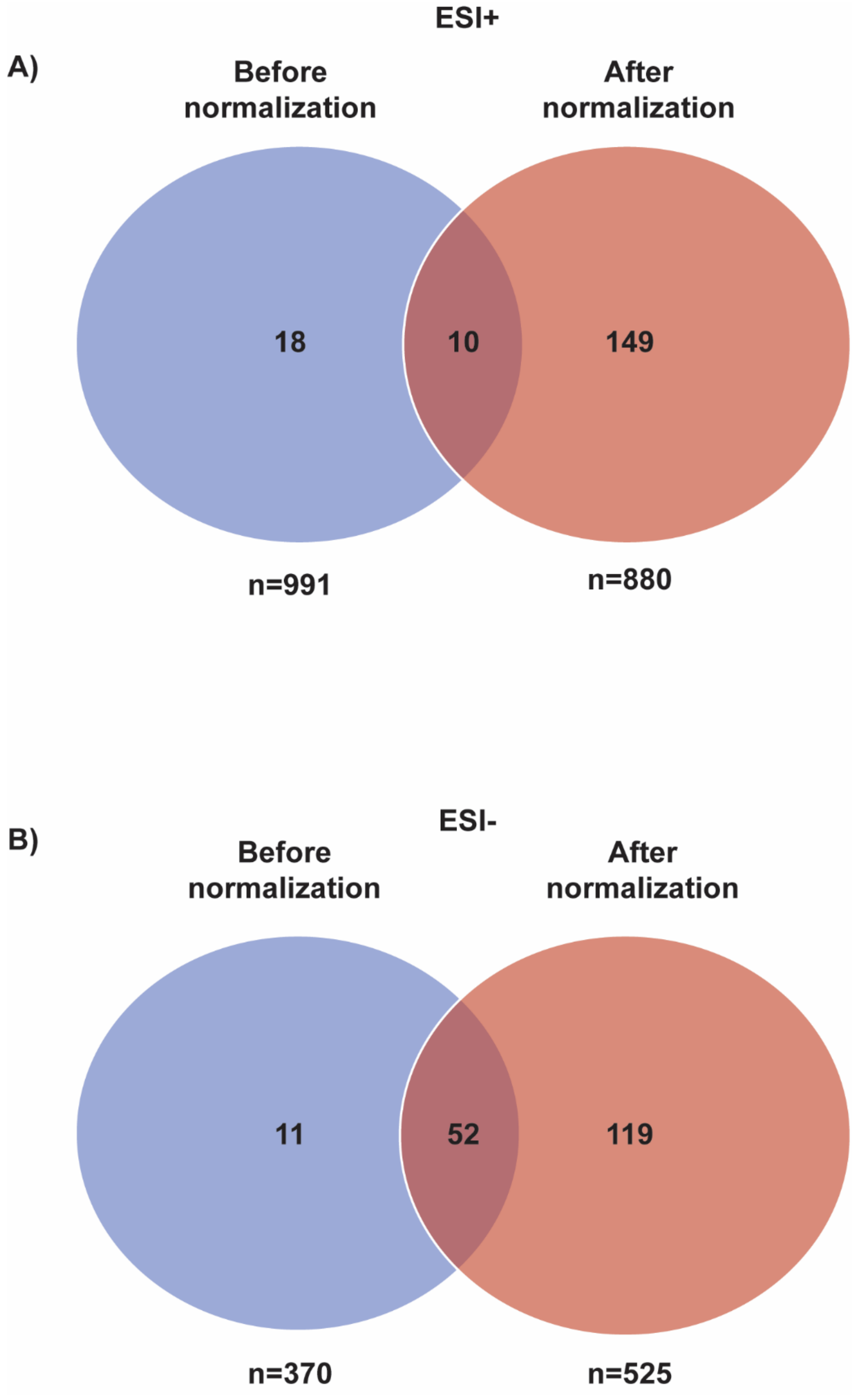

Figure S13. Venn diagram comparing the number of significant features between two groups of the clinical study before normalization (raw data) and after QC-SVRC + QC-norm normalization. Data from (A) ESI+ mode (B) ESI- mode. For statistics, t-test unpaired was used between groups.

Table S1. IS mix adducts detected in the experiment.

Compound	Adduct	*m/z*	RT (min)	Ionization mode
L-carnitine-D^3	$[M+H]^+$	165.1310	0.70	ESI+
	$[M+Na]^+$	187.1126		
Isoleucine- ^{13}C, ^{15}N	$(M+H)^+$	139.1184	0.77	ESI+
	$(M+Na)^+$	161.0992		
	$(M+K)^+$	177.0774		
	$[M-H]^-$	137.1044	0.77	ESI–
	$(M+Cl)^-[-H_2O]^-$	155.0748		
	$(M+Cl)^-$	173.0758		
Sphingosine-D^7	$(M+H)^+$	307.3343	14.38	ESI+
	$(M+Na)^+$	329.3156		
	$(M+K)^+$	345.2869		
LPC 18:1-D^7	$(M+H)^+$	529.3994	19.30	ESI+
	$(M+Na)^+$	551.3780		
	$(M+K)^+$	567.3681		
	$(M+H)^+$	529.4000	20.00	
	$(M+Na)^+$	551.3799		
	$(M+K)^+$	567.3525		
	$(M+Cl)^-[-H_2O]^-$	545.3464	19.30	ESI–
	$(M+Cl)^-$	563.3503		
	$(M+HCOO)^-$	573.3893		
	$(M-H)^-$	527.3535	20.00	
	$(M+HCOO)^-$ $[-H_2O]$	555.3490		
	$(M+Cl)^-$	563.3582		
	$(M+HCOO)^-$	573.3892		
Stearic acid-D^5	$(M-H)^-$	288.2955	34.4	ESI–
	$(M-H)^-$	289.2990		
	$(M-H)^-$	290.3026		

Note: RT = Retention time.

© 2019 by the authors. Submitted for possible open access publication under the terms and conditions of the Creative Commons Attribution (CC BY) license (http://creativecommons.org/licenses/by/4.0/).
